# Expression of the *Suaeda salsa SsNLP7* Transcription Factor in *Solanum lycopersicum* Enhances Its Salt Tolerance

**DOI:** 10.3390/plants15020175

**Published:** 2026-01-06

**Authors:** Cuijie Cui, Yan Chen, Xiaoyan Wu, Yi Xiong, Saisai Wang, Jianbo Zhu

**Affiliations:** 1 College of Life Sciences, Shihezi University, Shihezi 832000, China; ccj19987654321@163.com (C.C.); cy2524815698@163.com (Y.C.); wxy1245891567@163.com (X.W.); 2 Power China Eco-Environmental Group Co., Ltd., Shenzhen 518054, China; xyshzu2017@163.com

**Keywords:** *SsNLP7A*, salt tolerance, transcriptome, functional analysis, *Suaeda salsa*

## Abstract

The nitrate signaling core regulator *NLP7* is known to negatively regulate salt tolerance in *Arabidopsis thaliana*, but the function of the (*SsNLP7A*) gene in the halophyte *Suaeda salsa* remains unclear. To investigate whether *SsNLP7A* participates in salt stress responses, this study heterologously overexpressed the gene in tomato (*Solanum lycopersicum*) and systematically evaluated its function under salt stress through phenotypic, physiological, and transcriptomic analyses. The results indicate that *SsNLP7A* overexpression significantly promotes tomato root development and alleviates growth inhibition caused by salt stress. Under salt treatment, transgenic plants exhibited significantly higher chlorophyll content, accumulation of osmotic regulators (proline and soluble sugars), and antioxidant enzyme (POD, CAT, SOD) activity compared to wild-type plants. Transcriptome analysis further revealed that *SsNLP7A* enhances salt tolerance by regulating carbon metabolism, phytohormone signaling pathway, photosynthesis, and antioxidant pathways. Collectively, this study elucidates the positive regulatory role of *SsNLP7A* in salt stress response, providing new insights into its molecular mechanisms.

## 1. Introduction

Soil salinity is a major environmental stress that threatens the sustainability of global agriculture. It is estimated that over 20% of the world’s irrigated agricultural land is affected by salinity, leading to crop yield losses of 30–50% [1,2]. In general, appropriate nitrogen application generally enhances soil organic matter content and mitigates the inhibitory effects of salt on plant growth [3,4]. Hence, in regions with severe soil salinity, there is a practiced approach known as “using nitrogen to ameliorate salinity and alkalinity,” which involves applying nitrate- or ammonium-based nitrogen fertilizers to balance soil fertility and salt levels [5]. The main inorganic forms of nitrogen absorbed and utilized by plants are nitrate nitrogen (NO_3_^−^–N) and ammonium nitrogen (NH_4_^+^–N) [6,7].

Tomato (*Solanum lycopersicum* L.) is a major global food crop that is highly sensitive to salt stress, which severely inhibits its growth and reduces yield [8]. Salt stress not only impairs physiological processes through ion toxicity (primarily stemming from Na^+^ accumulation causing cellular ion imbalance and disruption of intercellular communication), but it also induces osmotic stress in specific cell types, triggers oxidative damage due to reactive oxygen species (ROS) imbalance across different cell types [9,10], and inhibits nitrogen uptake and assimilation by the root system, thereby exacerbating the decline in nitrogen use efficiency (NUE) [11]. Therefore, identifying key genes that can simultaneously enhance plant salt tolerance and NUE is crucial for advancing sustainable agriculture in saline environments.

*Suaeda salsa* is highly salt-tolerant and possesses a remarkable capacity for soil desalination. As a natural reservoir of salt-tolerance genes, investigating the molecular mechanisms underlying its response to salt stress holds great potential. Such research can not only enhance strategies for saline soil restoration but also facilitate the genetic improvement in crops by boosting their inherent salinity tolerance [12,13,14,15].

The transcription factor *NLP7* (NIN-like protein 7) serves as a central regulator of the primary nitrate response and is involved in plant adaptation to nitrogen deficiency and metabolism [16,17,18,19]. In *Arabidopsis*, *AtNLP7* enhances nitrogen assimilation and uptake by directly activating key genes such as the nitrate transporter *NRT1.1* and glutamine synthetase *GS1* [20]. Interestingly, *NLP7* gene function is also associated with carbon assimilation and salt tolerance. Under high salinity conditions, the application of nitrogen fertilizer promotes nitrogen and carbon assimilation through *AtNLP7*, thereby improving *Arabidopsis* growth performance. Moreover, *AtNLP7* overexpression promoted biomass accumulation, root development, and photosynthesis under varying nitrate conditions [21]. Its regulatory roles extend to light energy utilization (e.g., via the *NLP7*–*HB52*/54–*VAR2* pathway in rice; [22], reactive oxygen species (ROS) homeostasis mediated by HBI1; [23], stomatal opening, and lateral root development [24].

Notably, previous studies have shown that the loss-of-function of *AtNLP7* in *Arabidopsis* is negatively correlated with salt tolerance [25]. However, the relationship between *SsNLP7* in *Suaeda salsa* and plant salt tolerance remains unclear, making this study particularly significant.

This study aimed to investigate whether the *SsNLP7* gene from *Suaeda salsa* confers enhanced salt tolerance in tomatoes. The discovery and characterization of such resistance-related genes is crucial for crop improvement and the development of new resilient varieties, which has significant implications for sustainable agriculture and economic development.

## 2. Results

### 2.1. Bioinformatic Characterization of SsNLP7A Transcription Factor

**Figure 1:**
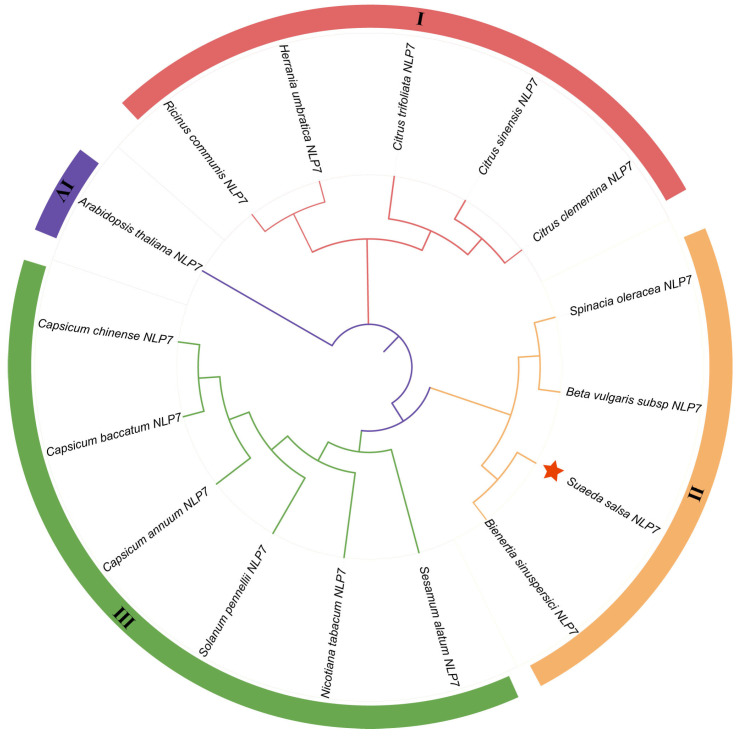
*SsNLP7A* phylogenetic tree analysis. Using MEGA6.0 software, a rootless phylogenetic tree of the *NLP7* protein sequences was constructed using the neighbor-joining method with 1000 bootstrap replicates. *SsNLP7A* is shown in red star.

The putative cDNA sequence encoding the *SsNLP7A* gene was isolated from *Suaeda salsa* leaves, and the full-length sequence was 2334 bp. The sequence contained a complete open reading frame (ORF) of 2233 bp, and *SsNLP7A* encoded 974 amino acids with a molecular mass of 86.04 kDa. We predicted the domain structures of the *SsNLP7A* protein and found that it contains a conserved RWP-RK located at amino acid positions 390–438 aa. The 679–759 aa contains another conserved domain PB1-*NLP*. The structure of these two conserved domains indicated that *SsNLP7A* is a member of the *NLP*s family (Figure S4). The protein consists of 27.72% alpha helix, 12.79% extended strand, 4.65% beta turn, and 52.84% random coil (Figure S5). The hydrophilicity and hydrophobicity of the proteins were analyzed using the ExPASy online tool (https://web.expasy.org/protscale/) (accessed on 1 January 2026). It is predicted that protein *SsNLP7A* is hydrophobic (Figure S6). An evolutionary tree was constructed using MEGA 6.0 using the maximum likelihood method, and the bootbp value was set to 1000 in order to obtain more reliable branches (Figure 1). Based on the phylogenetic relationship, the *SsNLP7A* gene is evolutionarily distant from the *AtNLP7* gene, implying that the gene functions may differ. The *SsNLP7A* gene is more closely branched and related to the *Bieneritia sinuspersici NLP7* gene relative to other species, and is evolutionarily recent. It was hypothesized that the genes have similar gene functions among their immediate homologues.

### 2.2. Relative Expression Level of SsNLP7A in Suaeda salsa

**Figure 2:**
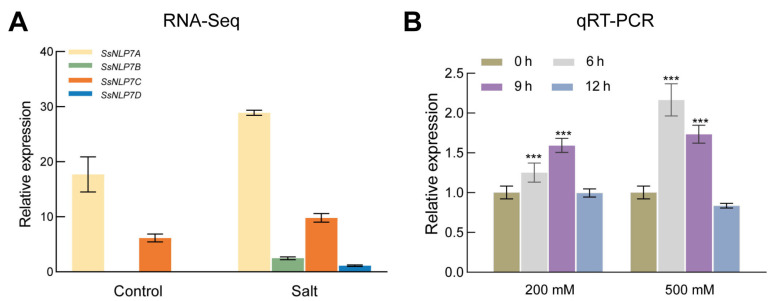
Expression of *SsNLP7A*, *SsNLP7B*, *SsNLP7C* and *SsNLP7D* genes during salt stress. (**A**) indicates the results of *SsNLP7A*, *SsNLP7B*, *SsNLP7C* and *SsNLP7D* transcriptome sequencing. (**B**) Expression of *SsNLP7A*, *SsNLP7B*, *SsNLP7C* and *SsNLP7D* genes in leaves of *Suaeda salsa* following treatment with 200 mM NaCl and 500 mM NaCl. Data are presented as the mean (±SD) of three biological replicates. Asterisks above the data bars indicate a significant difference (two-tailed *t*-test, *** *p* < 0.001) or no significant difference, respectively.

In publicly available transcriptomic data (The data can be found in the National Center for Biotechnology Information (NCBI) BioProject database under accession numbers PRJNA527358 and PRJNA512222.), we observed that all four *S. salsa NLP7* genes exhibited increased expression following salt stress, with *SsNLP7A* showing particularly significant (Figure 2A). To further analyze the relative expression levels of *SsNLP7A* in *S. salsa* under salinity stress, we collected leaves from plants treated with 200 mM and 500 mM NaCl at 0, 6, 9, and 12 h. qRT-PCR analysis revealed that *SsNLP7A* expression was significantly induced by salt stress (Figure 2B). Specifically, under 200 mM NaCl treatment, *SsNLP7A* transcript levels were upregulated at 6 and 9 h. In contrast, under 500 mM NaCl stress, its expression was rapidly induced at 6 h and remained at a high level throughout the time course. These results suggest that *SsNLP7A* plays an important role in the salt stress response of *S. salsa*.

### 2.3. Subcellular Localization of SsNLP7A Protein in Tobacco

**Figure 3:**
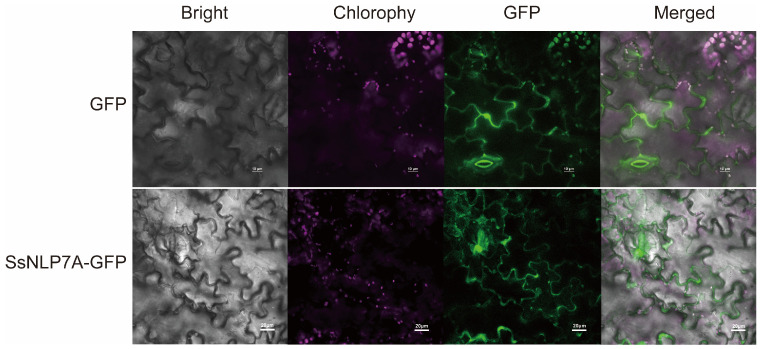
Subcellular localization of SsNLP7A-GFP fusion proteins.

According to predictions from the Cell-PLoc-2 online platform, SsNLP7A protein is localized to the cell nucleus (Figure S7). To experimentally determine its subcellular localization, we constructed a pCAMBIA1304-35S–*SsNLP7A*-sGFP fusion vector (Figure S6). The confirmed recombinant and control plasmids (pCAMBIA1304-35S-sGFP) were delivered into tobacco leaf cells via *Agrobacterium*-mediated transient transformation. Subcellular localization was observed using laser scanning confocal microscopy. The green fluorescence of the control GFP protein was distributed throughout the cell, including the plasma membrane, cytoplasm, stomata and nucleus. In contrast, the *SsNLP7A*-GFP fusion protein was specifically localized to the nucleus and cell membranes. These results indicate that *SsNLP7A* is a nucleo-membrane localized protein (Figure 3). This localization may vary in other cell types or plant species.

### 2.4. Identification of SsNLP7A-Overexpressing Tomato Plants

The identification of transgenic tomato plants was initially performed at the DNA level using PT-PCR technology. Total RNA was extracted from tomato seedling leaves, reverse-transcribed into cDNA, and amplified via PCR using *SsNLP7A* gene-specific primers (Table S1). The results were analyzed by 1% agarose gel electrophoresis. The results identified six transgenic tomato seedlings overexpressing the *SsNLP7A* gene (Figure S8A). To validate the transcriptional level of *SsNLP7A* in tomatoes, total RNA was extracted from leaves of these six positive seedlings. Following reverse transcription to prepare cDNA, semi-quantitative analysis was performed. The results (Figure S8B) revealed transcriptional activity of the *SsNLP7A* gene in only three seedlings. Subsequently, cDNA was extracted from these three positive seedlings and further validated via qRT-PCR, confirming significantly higher *SsNLP7A* expression levels in positive plants compared to wild-type (Figure S8C). Finally, PCR analysis using Agrobacterium Ti plasmid virB2 gene-specific primers confirmed that none of the three positive seedlings carried the virB2 gene. This yielded three tomato plants overexpressing the *SsNLP7A* gene (Figure S8D). All subsequent experiments utilized these three positive tomato plants.

### 2.5. Overexpression of SsNLP7A Promotes the Growth of Tomato

**Figure 4:**
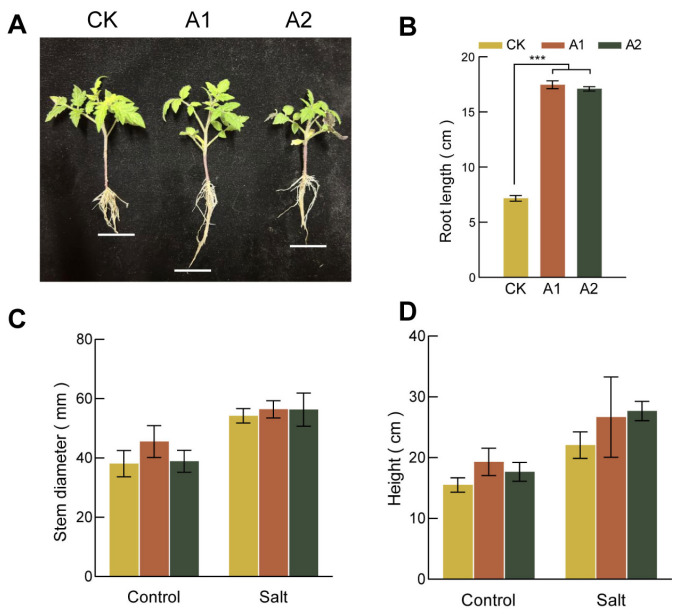
Growth of wild-type and transgenic tomato plants. (**A**) Root length of each strain under normal growth conditions. (**B**) Primary root length under normal growth conditions for each cultivar. (**C**) Plant height of each transgenic lines of tomato under salt stress. (**D**) Stem diameter of each strain under salt stress (*** *p* < 0.001 for comparisons between the transgenic lines and wild-type plants by Student’s *t*-tests).

Overexpression of *SsNLP7A* enhanced root development and salt tolerance in tomato. Under normal conditions, the root lengths of transgenic lines were significantly longer than those of wild-type (WT) plants (Figure 4A,B). Under normal conditions, there was no significant difference in stem thickness between the lines. However, under salt stress, the transgenic line A1 developed significantly thicker stems than the wild type (Figure 4C). Furthermore, after 20 days of salt treatment, the transgenic plants were significantly taller than the wild-type plants, which showed stunted growth (Figure 4D). Although the salt-induced increase in stem thickness was less dramatic than the increase in plant height, the transgenic plants ultimately maintained thicker stems than the wild-type controls under stress. Taken together, these results indicate that overexpression of *SsNLP7A* alleviates the inhibitory effects of salt stress on plant growth.

### 2.6. Overexpression of SsNLP7A Increases Tomato Salt Tolerance

**Figure 5:**
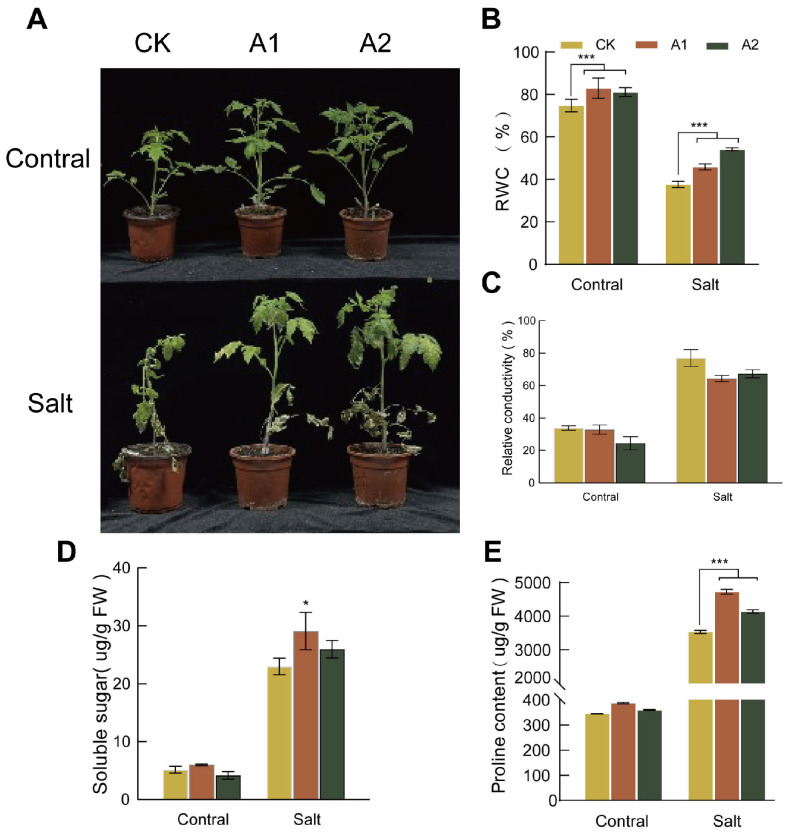
Phenotypic characteristics, relative water content, electrical conductivity, soluble sugar concentration, and proline concentration measurements in wild-type and transgenic tomato plants. (**A**) Phenotypic changes in wild-type and overexpressing *SsNLP7A* plants after 20 days of treatment with 300 mM NaCl. (**B**) Relative water content. (**C**) Relative conductivity. (**D**) Soluble protein. (**E**) Proline content. (* *p* < 0.05, *** *p* < 0.001 for comparisons between the transgenic lines and wild-type plants by Student’s *t*-test).

To assess salt tolerance, we treated both transgenic and wild-type plants with 300 mM NaCl for 20 days. The wild-type plants exhibited severe wilting and near-complete growth arrest. In contrast, transgenic plants overexpressing *SsNLP7A* showed only mild symptoms, such as leaf wilting and bending (Figure 5A). Prior to salt treatment, the RWC was comparable between transgenic and wild-type lines. As the treatment progressed, the RWC decreased in both groups; however, the transgenic lines maintained a significantly higher RWC than the wild-type lines (Figure 5B). Conversely, both transgenic and wild-type plants exhibited an increasing trend in relative electrical conductivity under prolonged stress. After 20 days of treatment, the relative electrical conductivity in wild-type plants was significantly higher than that in transgenic plants (Figure 5C). These findings indicate that overexpression of *SsNLP7A* effectively reduced membrane damage and improved water retention, thereby enhancing tolerance to salt stress.

Under normal conditions, no significant differences in proline or soluble sugar content were observed between the wild-type and transgenic lines. However, prolonged salt stress induced a continuous increase in both proline and soluble sugar levels in all plants. Notably, transgenic lines overexpressing *SsNLP7A* accumulated significantly higher levels of osmotic regulatory substances than wild-type controls. After 20 days of salt stress treatment, both soluble sugar and proline contents in transgenic plants were significantly higher than those in wild-type plants (Figure 5D,E). These results indicate that *SsNLP7A* overexpression promotes the accumulation of osmotic regulatory substances in plants, thereby enhancing salt tolerance.

### 2.7. Overexpression of SsNLP7A Enhances the Antioxidant Capacity of Transgenic Plants Under Salt Stress

**Figure 6:**
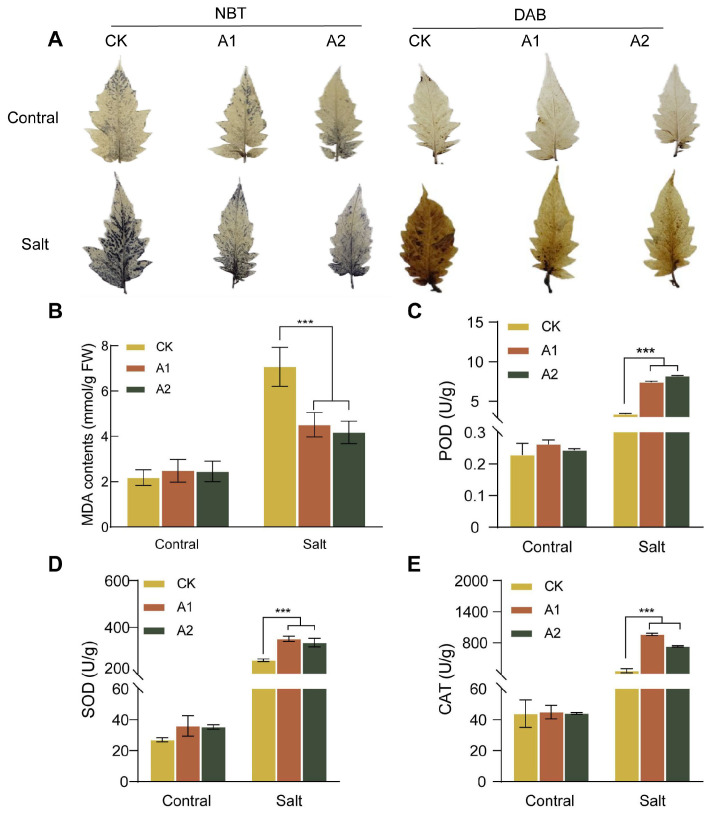
ROS production, MDA content, POD enzyme activity, SOD enzyme activity, and CAT enzyme activity in wild-type and transgenic tomato plants. (**A**) DAB staining and NBT staining. The darker color indicates greater ROS generates. (**B**) MDA content. (**C**) Peroxidase (POD) activity. (**D**) Superoxide Dismutase (SOD) activity. (**E**) Catalase (CAT) activity. (*** *p* < 0.001 for comparisons between the transgenic lines and wild-type plants by Student’s *t*-tests).

Salt stress triggers the overproduction of reactive oxygen species (ROS)—accumulation of H_2_O_2_, leading to oxidative damage in plant cells. MDA content, an indicator of general membrane lipid peroxidation, was lower in transgenic plants. Our results showed that under high-salt stress conditions (300 mM NaCl), the wild-type plants exhibited significantly higher MDA content than plants overexpressing *SsNLP7A* (Figure 6B). This result indicates that the transgenic plants experienced less membrane lipid peroxidation and therefore were subjected to less severe oxidative stress.

To further confirm the regulatory effect of *SsNLP7A* overexpression on ROS scavenging capacity, we examined the formation of ROS in transgenic and wild-type plants under salt treatment. As shown in the figure, no significant difference was observed between WT and *SsNLP7A*-overexpressing transgenic plants under normal conditions. However, after salt treatment, stronger histochemical staining was detected in WT leaves than in *SsNLP7A*-overexpressing transgenic plants (Figure 6A). In addition, several antioxidant enzymes play crucial roles in scavenging ROS under salt stress. To validate these findings, superoxide dismutase (SOD), peroxidase (SOD), and catalase (CAT) activities were measured. The results demonstrated that under high-salt conditions, the activities of POD, SOD, and CAT were significantly higher in transgenic tomato leaves than in WT (Figure 6C–E). These results indicate that overexpression of *SsNLP7A* can reduce ROS generates in plants, mitigate MDA-induced oxidative damage, and enhance tolerance in salt stress.

### 2.8. Overexpression of SsNLP7A Enhances the Photosynthesis of Transgenic Plants Under Salt Stress

**Figure 7:**
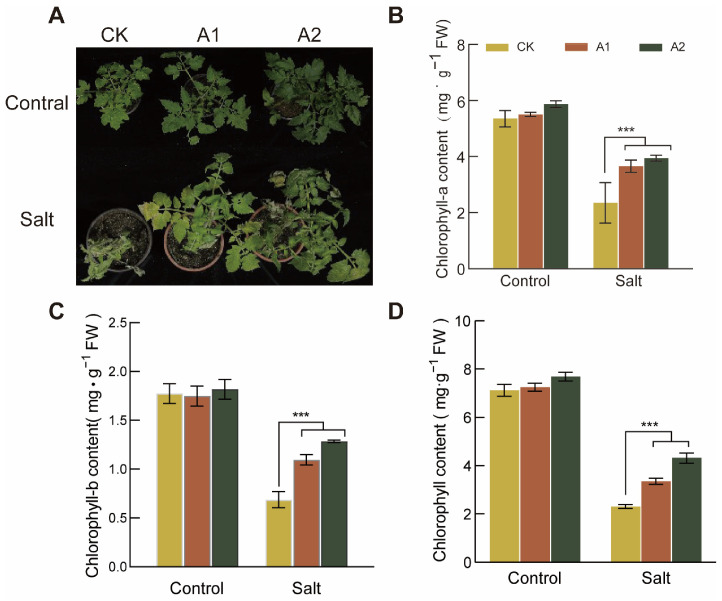
Determination of Chlorophyll Content in Wild-Type and Transgenic Tomato Plants. (**A**) Leaf chlorosis in each line of tomato after 20 days under salt stress (300 mM NaCl). (**B**) Chlorophyll-a content. (**C**) Chlorophyll-b content. (**D**) Chlorophyll content. (*** *p* < 0.001 for comparisons between the transgenic lines and wild-type plants by Student’s *t*-tests.).

To investigate the effect of salt stress on chlorophyll content in *SsNLP7A*-overexpressing and wild-type tomatoes, we measured leaf chlorophyll levels in both plant types. This study revealed no significant difference in chlorophyll content between transgenic and wild-type plants under normal conditions. Phenotypic observations after 20 days of high-salt stress treatment (300 mM NaCl) showed that wild-type plants exhibited more severe chlorosis than transgenic lines (Figure 7A).

Under normal growth conditions, no significant differences were detected in chlorophyll a, chlorophyll b, or total chlorophyll content among the lines. However, after 20 days of salt stress, the differences in chlorophyll a, chlorophyll b, and total chlorophyll reached their maximum. Under salt stress conditions, the chlorophyll a, chlorophyll b, and total chlorophyll contents in *SsNLP7A*-overexpressing tomatoes were significantly higher than those in the wild-type (Figure 7B–D). these findings indicate that *SsNLP7* enhances plant photosynthesis under high salt stress, thereby improving tomato salt tolerance.

### 2.9. Analysis of RNA Sequences and Identification of Differentially Expressed Genes in Tomato Under Salt Stress

The RNA sequencing (RNA-seq) results show that the cDNA libraries were created separately from the leaves taken at a concentration (300 mM) and grown for 60 days following high-salt stress, with three duplicates, and then sequenced on the Illumina Hiseq 2500 platform. After the initial sequencing readings have been checked for quality, 531138322 clean readings (6 Gb) were retained, but the Error Rate (Error Rate) level was kept low at 0.02 per cent for further analysis. The GC content was 42%, and the Q30 content was 95% (Table S2).

**Figure 8:**
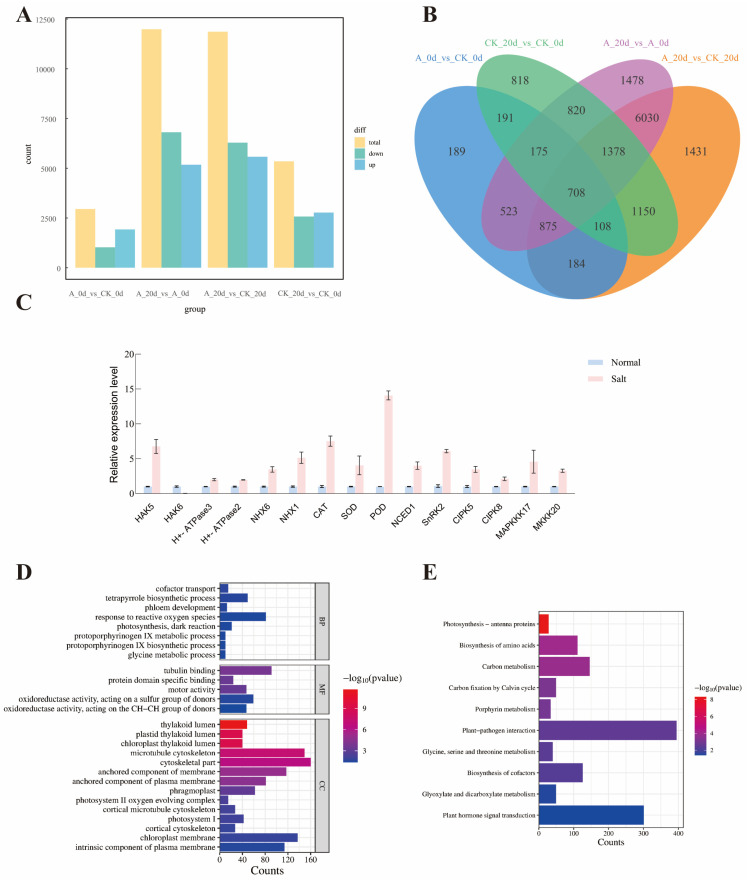
(**A**) Analysis of the number of differentially expressed genes between groups. (**B**) Intergroup differential gene Venn plot. (**C**) Expression analysis of relevant genes in transgenic and wild-type plants. Three independent biological replicates were performed (*n* = 3) (**D**) GO enrichment analysis. (**E**) KEGG enrichment analysis. (CK_0 d represents wild-type tomato plants under normal growth conditions; A_0 d represents transgenic tomato plants under normal growth conditions; CK_20 d represents wild-type tomato plants after 20 days of salt stress treatment; A_20 d represents transgenic tomato plants after 20 days of salt stress treatment.).

To analyze the impact of the *SsNLP7A* gene on tomato plants re-programming under salt stress, the number of differentially expressed genes (DEGs) between wild—type and the *SsNLP7A*-overexpressed transgenic tomato plants before and after high-salt stress was investigated. Firstly, a total of 5348 differentially expressed genes (DEGs) were identified in the first group. This group comprised wild-type tomato plants under normal growth conditions (CK_0 d) and wild-type tomato plants after 20 days of salt treatment (CK_20 d). In the second group, a total of 11,987 DEGs were detected between the *SsNLP7A*-overexpressed transgenic tomato plants under normal growth conditions (A_0 d) and the *SsNLP7A*-overexpressed transgenic tomato plants after 20 days of salt treatment (A_20 d) (Figure 8A). There were 3081 overlapping DEGs between the two groups, and these overlapping DEGs were caused by salt stress, which indicated that the young leaves of tomato rapidly reprogrammed the cellular response at the transcriptome level at salt stress (Figure 8B).

Among these two groups, 8906 DEGs were unique to the *SsNLP7A*-overexpressed transgenic tomato leaves before and after high-salt stress treatment (A_0 d and A_20 d) (Figure S9), indicating that the *SsNLP7A* gene induced a special effect in tomato plants under salt stress and altered the cellular responses at the transcriptome level of tomato plants.

The qRT-PCR analysis was carried out to validate the reliability of the RNAseq data. In the context of the transcriptome data, a selection of fifteen genes was identified within the A_0 d vs. A_20 d group that has been previously documented to be associated with salt tolerance in tomatoes, including HAK5, HAK6, H+-ATPase3, H+-ATPase2, NHX6, NHX1, CAT, SOD, POD, NCED1, SnRK2, CIPK5, CIPK8, MAPKKK17 and MKKK20 (Figure 8C). The qRT-PCR data showed that the expression of these genes in wild-type tomato plants were significantly lower than those in the *SsNLP7A*-overexpressed transgenic tomato plants under salt treatment, which was highly correlated (98.2%) with the transcriptome data (Figure S10).

### 2.10. GO and KEGG Enrichment Analysis of DEGs

After 20 days of high-salt treatment, the 8906 differentially expressed genes caused by the *SsNLP7A* gene were subjected to GO (Gene Ontology) functional annotation analysis according to three major categories: Biological process (BF), Molecular function (MF), and Cellular component (CC). These DEGs were significantly enriched (*p* ≤ 0.001) into 4007 GO terms, The terms “cofactor transport”, “tetrapyrrole biosynthetic process”, “phloem development”, “response to reactive oxygen species”, “photosynthesis, dark reaction”, “glycine metabolic process”, “protoporphyrinogen IX biosynthetic process”, and “protoporphyrinogen IX metabolic process”, were the dominant groups in the biological processes; “thylakoid lumen”, “chloroplast thylakoid lumen”, “plastid thylakoid lumen”, “microtubule cytoskeleton”, “cytoskeletal part”, “anchored component of membrane”, “anchored component of plasma membrane”, “phragmoplast”, “photosystem II oxygen evolving complex”, “cortical microtubule cytoskeleton”, “photosystem I”, “cortical cytoskeleton”, “chloroplast membrane” and “intrinsic component of plasma membrane” were the representative groups in the cellular components. Among the molecular functions, a great number of DEGs were focused on categories of “oxidoreductase activity, acting on the CH-CH group of donors”, “oxidoreductase activity, acting on a sulfur group of donors”, “motor activity”, “protein domain specific binding” and “tubulin binding” (Figure 8D).

To gain a deeper understanding of the reprogramming mechanism of the *SsNLP7A* gene in tomato plants under salt stress, we used KEGG pathways method to perform enrichment analysis. In this approach, a total of 10 routes were greatly enhanced. In particular, “Photosynthesis—antenna proteins”, “Biosynthesis of amino acids”, “Carbon metabolism”, and “Carbon fixation by Calvin cycle” were significantly enriched in most comparisons. The most genes were mainly related to stress response, indicating that *SsNLP7A* probably affected various stress-related genes in tomato to regulate salt tolerance. (Figure 8E).

**Figure 9:**
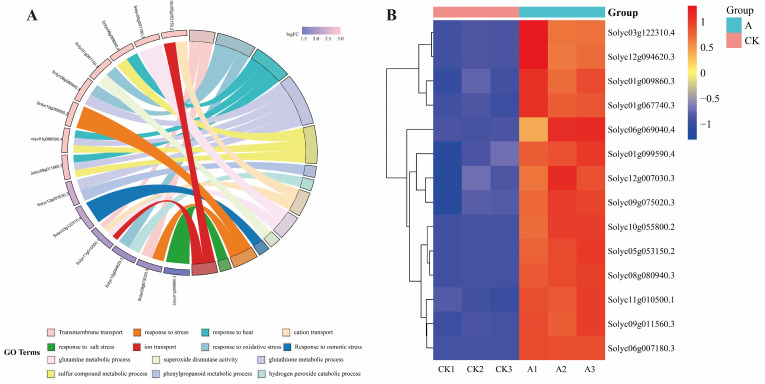
Analysis of 11 significantly expressed genes among differentially expressed genes associated with special effects caused by gene *SsNLP7A*. (**A**) GO enrichment analysis of differentially expressed genes. (**B**) Analysis of differential gene expression patterns. (CK1, CK2, CK3 represents wild-type tomato plants after 20 days of salt stress treatment; A1, A2, A3 represent transgenic tomato plants after 20 days of salt stress treatment).

From the 8906 differential genes, We identified 11 genes exhibiting significant expression changes, representing key functional pathways, which included the peroxidase-related genes Solyc08g080940.3, Solyc06g069040.4, Solyc01g099590.4, Solyc09g011560.3; ABC transporters gene Solyc09g075020.3; Phenylpropanoid biosynthesis gene Solyc12g007030.3; Peroxisome gene Solyc12g094620.3, Solyc05g053150.2; Arginine and proline metabolism gene Solyc03g122310.4; MAPK signaling pathway—plant gene Solyc10g055800.2; Corresponding stress-related genes Solyc01g009860.3, Solyc11g010500.1 and Solyc06g007180.3. The DEGs were significantly enriched into 14 GO terms, The terms involved Transmembrane transport, response to oxidative stress, response to heat, glutathione metabolic process, sulfur compound metabolic process, phenylpropanoid metabolic process, hydrogen peroxide catabolic process, cation transport, glutamine metabolic process, superoxide dismutase activity, Response to osmotic stress, response to stress and response to salt stress (Figure 9A). These genes were significantly induced by the *SsNLP7A* gene under salt stress (Figure 9B).

## 3. Discussion

This study reveals for the first time that overexpression of the halophyte *S. salsa* transcription factor gene *SsNLP7A* significantly enhances salt tolerance in tomato (*Solanum lycopersicum*). This finding contrasts with the classical understanding in *A*. *thaliana*, where *AtNLP7* negatively regulates salt tolerance. This functional divergence likely stems from *SsNLP7A*’s origin in a halophyte with extreme salt tolerance, which may have evolved distinct regulatory properties or interaction networks compared to model plants. Our integrated data indicate that *SsNLP7A* does not merely replicate the function of its *Arabidopsis* homolog, but instead plays a novel role as a positive regulator of salt tolerance in tomato by coordinating nitrogen metabolism with multiple stress response pathways.

Phenotypic analysis revealed that overexpression of *SsNLP7A* significantly promoted tomato root development, manifesting as longer roots and increased lateral root formation under normal conditions. This finding partially aligns with reports indicating that *AtNLP7* overexpression similarly enhances root growth in *Arabidopsis* [26], suggesting that its regulatory function in root development may be conserved across species. A well-developed root system serves as the fundamental structural basis for enhancing water and nutrient uptake. Moreover, under salt stress, *SsNLP7A* transgenic lines exhibited markedly superior growth compared to wild-type plants. As a core transcription factor in the primary nitrate response, *NLP7*′s function has been well-established in *Arabidopsis*, where it dominates nitrogen uptake by directly activating nitrate transport and assimilation genes such as *NRT1.1*. Therefore, we reasonably infer that *SsNLP7A* retains this core function in tomato. Nitrogen uptake is often suppressed under salt stress [27,28], and the sustained or enhanced expression of *SsNLP7A* may partially counteract this inhibition, ensuring continuous nitrogen supply. Adequate nitrogen nutrition not only provides raw materials for biomacromolecule synthesis but is also widely reported to mitigate stress-induced damage to the photosynthetic system [29,30,31,32,33,34,35], supporting chlorophyll synthesis and photosynthetic organ stability [36,37]. This aligns with our observation of higher chlorophyll content in transgenic plants under salt stress. Thus, *SsNLP7A* likely provides the essential material and energy prerequisites for plant salt stress response primarily through its inherent, conserved role in promoting nitrogen uptake and assimilation.

To investigate how *SsNLP7A* links enhanced nitrogen metabolism to salt tolerance phenotypes, we conducted transcriptomic analysis. Compared to wild-type plants subjected solely to salt stress, overexpressing plants exhibited 8906 differentially expressed genes that reflect better adaptation to salt in *SsNLP7A* overexpression lines. Enrichment analysis revealed how *SsNLP7A* systematically remodels stress response networks, both connecting with and expanding upon previous findings.

1. Synergy between hormone signaling and ion homeostasis

Differentially expressed genes were significantly enriched in plant hormone signaling pathways, with particularly prominent upregulation of ABA and auxin pathway genes (e.g., *SlABF2*, *SlPYL*, *SlAUX*/*IAA*). This aligns with numerous studies indicating ABA as the core hormone in salt stress responses, while auxin regulates root architecture [30]. Based on the findings of this study, we hypothesize that *SsNLP7A* may simultaneously enhance the regulatory functions of both signaling pathways. On one hand, activated ABA signaling may drive the expression of classical ion efflux systems like *SOS1* to maintain Na^+^ homeostasis [31]. On the other hand, enhanced auxin signaling could explain the observed root phenotypes. Crucially, recent studies reveal that ABA and auxin signaling also positively regulate *NRT1.1* [38]. Thus, *SsNLP7A* gene may occupy a regulatory hub where it simultaneously promotes ion homeostasis (salt tolerance) and nitrate uptake (nitrogen nutrition) through upregulating hormone signaling, achieving synergistic effects.

2. Carbon-nitrogen metabolic coupling and energy supply.

Carbon fixation and the upregulation of genes in carbon metabolic pathways indicate that *SsNLP7A* gene enhances carbon assimilation capacity. This aligns with Yu et al.’s finding that *AtNLP7* simultaneously boosts nitrogen and carbon assimilation. Under salt stress, this coupling proves critical: enhanced carbon metabolism provides essential carbon skeletons (e.g., α-ketoglutarate) for nitrate assimilation [39], while efficient nitrogen assimilation supplies nitrogen for photosystem repair and functional maintenance. This forms a positive feedback loop of “nitrogen metabolism-photosynthetic protection,” alleviating energy deficits induced by salt stress.

3. Enhanced antioxidant defense and redox homeostasis.

Upregulation of the peroxisome pathway and associated antioxidant enzyme genes (e.g., *SlCAT*, *SlPOD*) fully correlates with reduced ROS accumulation and elevated antioxidant enzyme activity in physiological data. Nitrogen serves as a key component for antioxidants like SOD and CAT; thus, *SsNLP7A*-driven nitrogen nutrition improvement likely directly enhances cellular antioxidant capacity [40,41,42]. Furthermore, the upregulation of glycine metabolism genes (*SlSHMT*) suggests potential regulation of the photorespiration pathway. Photorespiration functions as a safety valve under stress, and its product glycine serves as a precursor for the crucial antioxidant glutathione [43]. This provides an additional layer of explanation for the enhanced ROS scavenging capacity observed in the transgenic plants.

4. Potential associations with known regulatory networks.

We found that the protein kinase gene *SlCIPK8*, closely associated with nitrate signaling, was significantly upregulated in overexpressing plants. Studies by Hu et al. [44] and Marchive et al. [17] demonstrated that *CIPK8* positively regulates the low-affinity stage of the primary nitrate response in *Arabidopsis* and interacts with *NLP7*. *CIPK8* has also been shown to participate in regulating plant salt tolerance [45]. Therefore, we speculate that *SsNLP7A* gene may activate or synergize with *SlCIPK8* to jointly regulate downstream target genes. These downstream target genes likely include nitrate transporter genes, such as *SsNRT1.1*, which our laboratory previously identified as enhancing *Arabidopsis* salt tolerance [46]. This provides a plausible hypothesis for the regulatory pathway: *SsNLP7A* → (*SlCIPK8*) → *SlNRT1.1* → salt tolerance.

This study first establishes a positive correlation between *SsNLP7A* from halophytes and crop salt tolerance, challenging the established understanding that *AtNLP7* negatively regulates salt tolerance in Arabidopsis. It provides a novel paradigm for utilizing genetic resources from plants adapted to extreme environments. Through integrated analysis, we propose that *SsNLP7A* enhances salt tolerance by coordinating the “nitrogen metabolism-hormone signaling-ion homeostasis” network. This deepens our understanding of the cross-talk mechanisms between nutritional and environmental signals in plant stress responses. It is important to note that the DEGs identified here represent a transcriptional readout of the adaptive state in SsNLP7A-overexpressing plants under prolonged salt stress. While they highlight key biological processes involved in this adaptation (e.g., enhanced antioxidant capacity and photosynthesis), they correlate with but do not definitively establish the mechanistic causality. This core mechanism likely originates from the primary function of *SsNLP7A* as a transcription factor, which directly or indirectly regulates the expression of a series of downstream genes to establish a new steady-state equilibrium. Subsequent studies will employ in situ time-series analysis of cell geometry, chromatin, and protein activity to see how transgenic plants reach new balance between cell types under new conditions.

## 4. Materials and Methods

### 4.1. Plant Materials

Seeds of *Suaeda salsa* were collected in Gansu Province and subsequently stored in the laboratory of Professor Zhu Jianbo at Shihezi University, Xinjiang, China. The seeds underwent disinfection. First, they were disinfected with 75% ethanol for 30 s and then rinsed. Following this, surface disinfection was performed using a 15% sodium hypochlorite solution for 8 min. The seeds were then inoculated onto solid Murashige-Skoog (MS) medium (purchased from a supplier in Beijing, China) for cultivation. Wild-type tomato (*Solanum lycopersicum* L.) seeds of the ‘Yaxin 87-5’ cultivar were provided by Yaxin Seed Industry Co., Ltd. (Shihezi City, China). Tomato seeds were similarly disinfected and then inoculated onto solid Murashige-Skoog (MS) medium.

### 4.2. Search for SsNLP7A and Screening in Suaeda salsa

To investigate *SsNLP7* homologs in *Suaeda salsa*, published transcriptome data under salt stress (300 mM NaCl) were downloaded from the NCBI SRA database (https://www.ncbi.nlm.nih.gov/bioproject?term=PRJNA527358&cmd=DetailsSearch) (accessed on 1 January 2026). Raw sequencing reads were processed using FASTP v. 0.20.0 to remove low-quality bases and adapter sequences, yielding clean data for subsequent analyses. Quality metrics, including total bases, total reads, Q20, Q30, GC content, and valid data ratios, were calculated. The overall data quality was assessed using FastQC (Babraham Bioinformatics—FastQC A Quality Control tool for High Throughput Sequence Data (https://www.bioinformatics.babraham.ac.uk/projects/fastqc/) (accessed on 1 January 2026). De novo transcriptome assembly was performed on the cleaned reads using Trinity v. 2.9.1. The longest transcript from each gene cluster was selected as a unigene to represent the putative sequences. The coding sequence (CDS) of *AtNLP7* (AT4G24020) from *A. thaliana* was retrieved from GenBank (https://www.ncbi.nlm.nih.gov/gene/?term=AT4G24020) (accessed on 1 January 2026) and used as a query for local BLAST analysis of the assembled *Suaeda salsa* unigene database. A TBLASTN (https://blast.ncbi.nlm.nih.gov/Blast.cgi) (accessed on 1 January 2026) search was conducted with a maximum E-value cutoff of 1 × 10^−10^, and other parameters were set to default. Candidate sequences identified from the BLAST results were extracted, and duplicates were eliminated. Initial sequence comparison and alignment were conducted using DNAMAN. The putative *S. salsa NLP7* nucleotide sequences were translated into amino acid sequences using DNAMAN (DNAMAN—Lynnon Biosoft Bioinformatic Solutions (https://www.lynnon.com/dnaman.html) (accessed on 1 January 2026). These protein sequences were subjected to homology analysis using online BLASTP and multiple sequence alignment. Conserved protein domains were identified by searching the translated sequences against the NCBI Conserved Domains Database (CDD) (https://www.ncbi.nlm.nih.gov/cdd) (accessed on 1 January 2026). The tertiary structure of SsNLP7A was predicted using the SWISS-MODEL online server. Physicochemical properties, including hydrophobicity, were analyzed using the ProtScale tool on the ExPASy server (https://web.expasy.org/protscale/) (accessed on 1 January 2026). A phylogenetic tree was constructed to determine the evolutionary relationships. Homologous protein sequences from various species were retrieved using NCBI BLASTP (https://www.ncbi.nlm.nih.gov/Structure/cdd/wrpsb.cgi) (accessed on 1 January 2026). Multiple sequence alignments were performed using MEGA (https://mega.nz/login) (accessed on 1 January 2026), and a neighbor-joining (NJ) phylogenetic tree was constructed with 1000 bootstrap replicates.

### 4.3. Gene Expression Analysis

Wild-type *S. salsa* seedlings grown to 60 days old were treated with 0, 200, or 500 mM NaCl solutions for 0, 6, 9, and 12 h. Leaves were harvested from the same node of *S. salsa* plants under different salt concentrations and treatment durations, with three biological replicates per group. Total RNA was extracted from wild-type ‘Yaxin 87-5’ tomato leaves using the OMEGA Difficult Plant RNA Extraction Kit (R4165-02) (Originally from the United States, supplied by Shanghai Cent Bio). Subsequently, the extracted RNA was reverse-transcribed into first-strand cDNA using the TransScript^®^ One-Step gDNA Removal and cDNA Synthesis SuperMix kit (Purchased from Beijing TransGene Biotech), following the manufacturer’s protocol. Gene-specific primers for *SsNLP7A*, *SsNLP7B*, *SsNLP7C*, and *SsNLP7D* were designed using Primer5 software (Primer Premier: Software for PCR Primer Design|Primer Design Program (premierbiosoft.com) (https://www.premierbiosoft.com/primerdesign/) (Accessed on 1 January 2026). The relative transcript abundance of these genes in the leaves of wild-type *S. salsa* was determined using quantitative real-time PCR (qRT-PCR). The qRT-PCR reaction system in this experiment was 20 µL. Before the experiment, the cDNA concentration of each material was detected using NanoDrop spectrophotometry (Thermo Fisher, Waltham, MA, USA) and a standard of 600 ng of cDNA was uniformly added. The remaining volume was supplemented with ddH_2_O. *SsACTIN* was selected as the internal reference gene. The qRT-PCR reaction system is in Table 1. The primer sequences used are listed in Supplementary Materials (Table S1).

### 4.4. Cloning of SsNLP7A and Construction of Plant Expression Vectors

Total RNA was extracted from *S. salsa* leaves using the RNAiso Plus kit (TaKaRa, Biomedical Technology (Beijing) Co., Ltd., Beijing, China) according to the manufacturer’s instructions. Subsequently, cDNA was synthesized from the extracted RNA using a reverse transcription kit (TaKaRa, Biomedical Technology (Beijing) Co., Ltd., Beijing, China). Gene-specific primers were designed based on the *SsNLP7A* sequence using Primer 5.0, *KpnI* and *Sam I*’s restriction sites were supplemented with *SsNLP7A* (Table S1). The cDNA served as a template for PCR amplification to generate the *SsNLP7A* fragment. The PCR reaction system is in Table 2. The PCR products were purified, analyzed by agarose gel electrophoresis, and then cloned into the pMD19-T vector. Transform the constructed plasmid into *E*. coli DH5α competent cells using the heat shock method. Positive clones were screened and verified by DNA sequencing. Finally, the confirmed *SsNLP7A* fragment was ligated into the plant expression vector pCAMBIA2300 via the *BamH I* and *Sma I* restriction sites. The recombinant plasmid was introduced into *Agrobacterium tumefaciens* GV3101 using the freeze–thaw transformation method. Positive colonies were identified and cultured for further experiments (Figure S1). All primer sequences used in this study are listed in the Supplementary Materials (Table S1).

### 4.5. Transformation of Tomato and Acquisition of Transgenic Positive Lines

This study employed *Agrobacterium*-mediated genetic transformation to generate positive transgenic tomato lines using hypocotyl explants were obtained from 7-day-old wild-type tomato seedlings grown under sterile conditions. The transformation procedure was conducted as follows: hypocotyls were aseptically cut into approximately 1 cm^2^ segments and placed in Petri dishes containing MS medium (Murashige and Skoog Medium, a commonly used basal medium in plant tissue culture). for pre-culture. The dishes were then incubated in darkness for 36 h. An *Agrobacterium tumefaciens* culture harboring the target gene was activated (OD_600_ value of 0.6–0.8), centrifuged to pellet the bacterial cells, and resuspended in liquid MS medium. The hypocotyl explants were immersed in this bacterial suspension for 20 min. After incubation, the explants were blotted dry on sterile filter paper to remove excess bacteria and transferred onto fresh MS medium for a 48 h co-cultivation period in the dark. Following co-cultivation, the hypocotyls were transferred to a selection medium (MS basal medium supplemented with 2.0 mg/L 6-BA, 0.5 mg/L NAA, and 50 µg/mL kanamycin) to induce shoot formation (Figure S2). MS medium, sucrose, and agar were purchased from Sangon Biotech Co., Ltd. (Shanghai, China). Regenerated shoots measuring 3–5 cm in length with multiple branches, moderate root hair development, and a robust root system were selected. These plantlets were acclimatized under high light conditions in sealed containers for 15 days before being transplanted into a mixed substrate (peat soil: vermiculite: perlite = 3:1:2, *v*/*v*/*v*). Transgenic tomato plants were maintained in a growing room at 25–28 °C, 65–70% humidity, 8000 lx light intensity, and a 16/8 h light/dark cycle. During the initial transplanting period, cover plants with paper cups to reduce water evaporation and regularly mist to maintain high humidity. Plants suspected to be transgenic and wild-type control plants surviving 40 days post-transplanting were selected for molecular validation. Tomato plant cDNA was extracted and analyzed via RT-PCR. Wild-type tomato plants served as negative controls, while the *SsNLP7A* gene plasmid acted as a positive control. Identified positive plants underwent semi-quantitative analysis for further screening. Subsequently, qRT-PCR experiments were conducted to identify positive plants. Finally, positive tomato plants were confirmed via PCR detection (using specific primers targeting the virB2 gene on the Agrobacterium Ti plasmid) and field-transplanted to cultivate T2 generation plants to obtain homozygous transgenic seeds. Subsequent experiments utilized 60-day-old tomato seedlings grown from potted T2 generation seeds. Supplementary Materials are detailed in (Table S1, Figure S8).

### 4.6. Subcellular Localization Analysis of SsNLP7A

The subcellular localization of the protein was predicted using the online tool Cell-PLoc 2.0 (Cell-PLoc 2.0 package (sjtu.edu.cn) (http://www.csbio.sjtu.edu.cn/bioinf/Cell-PLoc-2/) (accessed on 1 January 2026). To experimentally validate this prediction, the coding sequence of *SsNLP7A* was amplified by high-fidelity PCR using gene-specific primers, *SsNLP7A*-F2 and *SsNLP7A*-R2, which incorporated *BamH I* and *Sbf I* restriction sites. The amplified product was subsequently cloned into the GFP-fusion expression vector pCAMBIA1304 under the control of the 35S promoter to generate the recombinant plasmid pCAMBIA1304-35S::GFP-*SsNLP7A*. Both constructs and the empty vector control (pCAMBIA1304-35S::GFP) were introduced into *A. tumefaciens* strain GV3101. The bacterial suspensions were then infiltrated into the leaves of *Nicotiana benthamiana*. After incubating the plants under normal growth conditions for 48 h, the epidermal layers of the infiltrated leaves were examined for GFP fluorescence using laser scanning confocal microscopy (Figure S3).

### 4.7. Experimental Treatment

For phenotypic analysis, 60-day-old wild-type and transgenic tomato plants (grown under standard room temperature conditions) were treated with 300 mM NaCl for 20 days. Three independent transgenic lines were included in the stress assay, with the wild type serving as the control. Each group consisted of three biological replicates each. Plant growth was monitored throughout the treatment period, and photographs were taken to document the phenotypic differences.

### 4.8. DAB Staining and NBT Staining

Solutions of 1 mg/mL diaminobenzidine (DAB) (Beijing Solarbio Science & Technology Co., Ltd., Beijing, China) and 0.5 mg/mL nitroblue tetrazolium (NBT) (Beijing Solarbio Science & Technology Co., Ltd., Beijing, China) were prepared using 0.01 mM phosphate buffer, pH 7.2. Leaves from the same nodal position of wild-type and transgenic tomato plants, after being treated for 20 days with either water or 300 mM NaCl, were collected and placed individually in 50 mL centrifuge tubes. The leaves were completely submerged in the respective staining solution (DAB or NBT) and incubated in the dark until characteristic staining patterns emerged: blue precipitate formation for NBT (indicating superoxide accumulation) and brown polymerization products for DAB (indicating the presence of hydrogen peroxide). After staining, the leaves were decolorized in 95% ethanol in a boiling water bath for 5 min to remove chlorophyll. The samples were then photographed for documentation and analyzed further.

### 4.9. Measurement of Physiological and Biochemical Indicators

The experiment selected uniformly grown 60-day-old plants (at 25–28 °C, 65–70% humidity, 8000 lx light intensity, and a 16/8 h light/dark cycle). To induce stress, 150 mL of a 300 mM NaCl solution was applied to wild-type and transgenic tomato plants every three days starting from day 1, continuing until day 20. After treatment, the leaf was harvested from each plant. Leaf relative water content (RWC) was determined by weighing. Leaves were immersed in distilled water for 24 h to record saturated fresh weight (TW); subsequently, leaves were oven-dried to determine dry weight (DW). RWC (%) was calculated using the following formula: RWC (%) = [(FW − DW)/(TW − DW)] × 100. Electrolyte leakage rate was measured using an EC215 conductivity meter (Markson Science Inc., Henderson, NC, USA) [47]. After leaf immersion in distilled water for 12 h, the initial conductivity of the leaf leachate (L_1_) was measured. The same sample was subjected to 30 min of boiling water treatment, after which the final conductivity (L_2_) was measured. The relative conductivity was calculated using the formula: REL (%) = (L_1_/L_2_) × 100. Malondialdehyde (MDA) content was determined using the thiobarbituric acid (TBA) method [48]. Weigh 0.1 g of fresh leaves, add 400 μL of liquid nitrogen grinding solution, and centrifuge at 6000 rpm for 15 min. Transfer 400 μL of supernatant to a new centrifuge tube, add 0.5% thiobarbituric acid solution dissolved in 5% trichloroacetic acid, then quantitatively add 1 mL of trichloroacetic acid. Mix thoroughly, cool to room temperature on ice, and centrifuge at 5000 rpm for 15 min. Mix thoroughly again, cool to room temperature on ice, centrifuge at 5000 rpm for 15 min, and measure the absorbance at 600 nm. Subsequently, immerse the sample in boiling water for 30 min. Glutaraldehyde concentration C (μmol/L) = 6.45 × (A_532_ − A_600_) − 0.56 × A_450_. Absorbance values were measured using a UV1901PC UV-Vis spectrophotometer (Shanghai Aoxi Scientific Instrument Co., Ltd., Shanghai, China). Proline content was determined using the urea salicylic acid method [49]. The mixture was heated for 15 min and then filtered. The filtrate was placed in a boiling water bath for 30 min, followed by the addition of 2 mL of 2.5% acidic indophenol solution and 2 mL of glacial acetic acid. Four milliliters of toluene were added, and the mixture was thoroughly shaken. After cooling, the layers separated. The upper layer was transferred to a new 10 mL centrifuge tube and centrifuged at 3000 rpm for 5 min. Compare the absorbance value against the previously determined proline standard curve. Soluble sugars are determined using the anthrone colorimetric method [50]. Mix 0.1 g of fresh leaves with 10 mL of distilled water, boil for 30 min, cool to room temperature, and dilute to 100 mL. Add 1 mL of the sample to 5 mL of 0.2% anthraquinone solution and measure the absorbance at 625 nm, comparing it to the glucose standard curve. Chlorophyll and carotenoid content were determined spectrophotometrically using a modified Arnonmethod [22]. Take 0.1 g of leaf tissue from plants before and after treatment, cut into small segments, and immerse in 25 mL of 95% (*v*/*v*) ethanol. The tubes were placed in a dark environment at room temperature until the leaf tissue was completely bleached. The absorbance of the extract was measured at wavelengths of 470, 649, and 665 nm, with 95% ethanol serving as the blank control. The concentrations of chlorophyll a, chlorophyll b, and total carotenoids in the extract were calculated using the following formula: Ca (mg/L) = 13.95 × A_665_ − 6.88 × A_649_; Cb (mg/L) = 24.96 × A_649_ − 7.32 × A_665_. The activities of peroxidase (POD), superoxide dismutase (SOD), and catalase (CAT) were measured using commercial assay kits (BC0090 for POD, BC0170 for SOD, and corresponding kit for CAT, Solebio, Beijing, China) which detects both cytoplasmic and plastidic isoforms, according to the manufacturer’s protocols.

### 4.10. RNA-Sequencing Data Analysis

First, plant materials and growth conditions: T3-generation tomato plants overexpressing the *SsNLP7A* gene were co-cultured with the wild-type (WT) variety “Yaxin 87-5” for 60 days under constant temperature (25 °C) and a photoperiod of 8 h light/16 h dark. Second, salt stress treatment and sampling: Plants with similar growth vigor were selected for the experiment. The treatment group was irrigated with 300 mM NaCl solution for 20 days, while the control group received distilled water. Four experimental groups were established: wild-type control (CK_0 d), overexpression control (A_0 d), wild-type after 20 days of salt stress (CK_20 d), and overexpression group after 20 days of salt stress (A_20 d). Each group comprised three biological replicates. Leaf tissue samples were collected from all plants at the start (0 days) and end (20 days) of treatment. All samples were immediately immersed in liquid nitrogen for subsequent transcriptome data analysis. The sample transcriptome sequencing was performed by Wuhan Maiwei Metabolic Biotechnology Co., Ltd. (Wuhan, China).

### 4.11. Data Processing and Analysing

Preliminary data statistics were performed using Excel, and the correlation coefficient was analyzed, and the fitting parameters of the logistic equation were obtained using SPSS statistics 18.0 software (https://spss.mairuan.com/) (accessed on 1 January 2026). The membership function values of the physiological indexes were calculated and evaluated using the membership function method. Calculation of the membership function values:

U(Xj) = (X − Xmin) (Xmax − Xmin) OR U(Xj) = 1 − [(X − Xmin) (Xmax − Xmin)] Among them: ’j’ = 1, 2, 3, … n. ’Xj’ represents the measurement value of the ’j-th’ factor. ’Xmax’ and ’Xmin’ are the maximum and minimum values of the measured values for this indicator, respectively. Figures were drawn using GraphPad Prism 8.3 software. We used Student’s *t*-test to indicate significance. * *p* < 0.05, ** *p* < 0.01, *** *p* < 0.001, and **** *p* < 0.0001.

## Figures and Tables

**Table 1:** qRT-PCR reaction system (F: Forward primers; R: Reverse primers).

Component	Volume (µL)
cDNA	3–5
2 × qPCR SuperMix	10
Primer-F (10 µM)	1
Primer-R (10 µM)	1
ddH_2_O	3–5

The qRT-PCR amplification reaction program included predenaturation at 94 °C for 3 min, denaturation at 94 °C for 15 s, annealing at 58 °C for 15 s and extension at 72 °C for 10 s. There were 40 cycles of denaturation, annealing and extension. The 2^−∆∆T^ method was used to analyze the experimental qRT-PCR data.

**Table 2:** PCR reaction system (F: Forward primers; R: Reverse primers).

Component	Volume (µL)
cDNA	1–2
2 × PCR SuperMix	10
Primer-F (10 µM)	1
Primer-R (10 µM)	1
ddH_2_O	6–7

The PCR amplification reaction program included predenaturation at 95 °C for 5 min, denaturation at 95 °C for 15 s, annealing at 58 °C for 30 s and extension at 72 °C for 10 s. There were 35 cycles of denaturation, annealing and extension.

## Data Availability

The original contributions presented in this study are included in the article/Supplementary Material. Further inquiries can be directed to the corresponding authors.

## Supplementary Materials

The following supporting information can be downloaded at: https://www.mdpi.com/article/10.3390/plants15020175/s1. Figure S1. (A) Cloning the SsNLP7A gene from Suaeda salsa. Numbers 1–2 indicate the SsNLP7A gene cloned from Suaeda salsa’s cDNA. M denotes Maker, CK denotes negative control. (B) SsNLP7A overexpression vector map. (C) The identification of recombinant plasmids in plant expression vectors using enzyme digestion. Number 1–4 indicates the use of Kpn I, Sal I and Sal I enzymes to digest the pCAMBIA2300-SsNLP7A recombinant plasmid. “+” indicates the plasmid that has not been digested by Kpn I and Sal I double enzymes. “M” indicates the marker (Maker III). (D) Identification of Agrobacterium tumefaciens carrying the transformed pCAMBIA2300-SsNLP7A recombinant plasmid by PCR. Numbers 1–9 represent the monoclonal samples that were tested. “+” indicates the positive control, “─” indicates the negative control. “M” indicates the marker (Maker III). Figure S2. The Genetic transformation process of Solanum lycopersicum. (B) Callus induced from hypocotyl on MS medium supplemented with 2.0 mg/L 6-BA and 0.5 mg/L NAA. (C) Further differentiation of callus into Solanum lycopersicum callus-derived plantlets. (D) Transfer the callus-derived plants to bottles containing culture medium for cultivation. (E) Rooting induction on 1/2MS medium supplemented with 0.5 mg/L IAA. (F) Development of complete plants after transplantation (Pot: 7.2 cm inner diameter, 11.5 cm high). Bar = 2.5 cm. (G) Transplant plants to the field for cultivation and collect seeds. (H) Harvest T2 generation seeds for laboratory cultivation to prepare for subsequent experiments. Figure S3. Construction of transient expression vectors of SsNLP7A. (A) Cloning the GFP gene from pCAMBIA1304 vector. Numbers 1–2 indicate the GFP gene cloned from pCAMBIA1304 plasmid. (B,C) The identification of recombinant plasmids in plant expression vectors using enzyme digestion. Number 1–4 indicate the use of BamH I and Kpn I enzymes to digest the pCAMBIA2300-SsNLP7A-GFP recombinant plasmid. “+” indicates the plasmid without the addition of BamH I and Kpn I as a control, and “M” indicates the marker (Maker III). (D) Identification of Agrobacterium tumefaciens carrying the transformed recombinant plasmid by PCR. Numbers 1–10 represent the monoclonal samples of Agrobacterium tumefaciens transformed with pCAMBIA2300-SsNLP7A-GFP. “+” indicates the positive control, “─” indicates the negative control. “M” indicates the marker (Maker III). Figure S4. *SsNLP7A* sequence structure analysis. Multiple sequence alignment of *SsNLP7A* and NLP7s aquaporin from *Arabidopsis thaliana*. Figure S5. Secondary Structure Prediction of SsNLP7A Protein. Figure S6. SsNLP7A Hydrophilicity/Hydrophobicity Analysis. Figure S7. Subcellular Localization Prediction for SsNLP7A. Figure S8. Identification of tomato plants overexpressing the *SsNLP7A* gene. (A) Characterization of SsNLP7A transgenic hybrid *Solanum lycopersicum* through DNA-PCR. A total of 15 *Solanum lycopersicum* plants were identified, with 6 plants overexpressing the SsNLP7A gene confirmed as positive. These were designated as OE1, OE2, OE3, OE4, OE5, and OE6. WT represents the wild-type tomato plant. (B) Characterization of SsNLP7A transgenic hybrid *Solanum lycopersicum* through RNA-PCR. Six positive plants identified at the DNA-PCR were subjected to RNA-PCR identification. A total of three positive plants were identified: OE4, OE5, and OE6. WT represents the wild-type *Solanum lycopersicum* plant. (C) cDNA was extracted from leaves of three positive transgenic plants identified from Figure B, alongside cDNA from wild-type tomato leaves. qRT-PCR experiments were conducted using primers listed in Table S1, with wild-type tomato plants serving as controls. The housekeeping gene used is: SlGAPDH. (D) Using genomic DNA from T2-generation transgenic plants, PCR detection was performed with primers specific for the virB2 gene on the *Agrobacterium* Ti plasmid. No specific band for the virB2 gene was amplified in DNA samples from all three transgenic lines (OE1, OE2, OE3) and the wild-type (WT) control, whereas the positive control (Agrobacterium GV3101 genomic DNA) showed a clearly visible band. Figure S9. Venn diagram of differentially expressed genes between A_0d VS A_20d and CK_0d VS CK_20d groups. Figure S10. Consistency between RNA-seq and qRT-PCR in detecting fold change (FC) in gene expression. Table S1. Primer sequences. Table S2. Sample.
