# Supplementary material for: Expression of the Suaeda salsa SsNLP7 Transcription Factor in Solanum lycopersicum Enhances Its Salt Tolerance
Source: Plants (Basel). 2026 Jan 6;15(2):175. doi: 10.3390/plants15020175 (PMC12845441; doi:10.3390/plants15020175)
Supplement: Supplementary file 1 [file plants-15-00175-s001.zip › plants-4025721-supplementary.pdf]

Supplementary Material

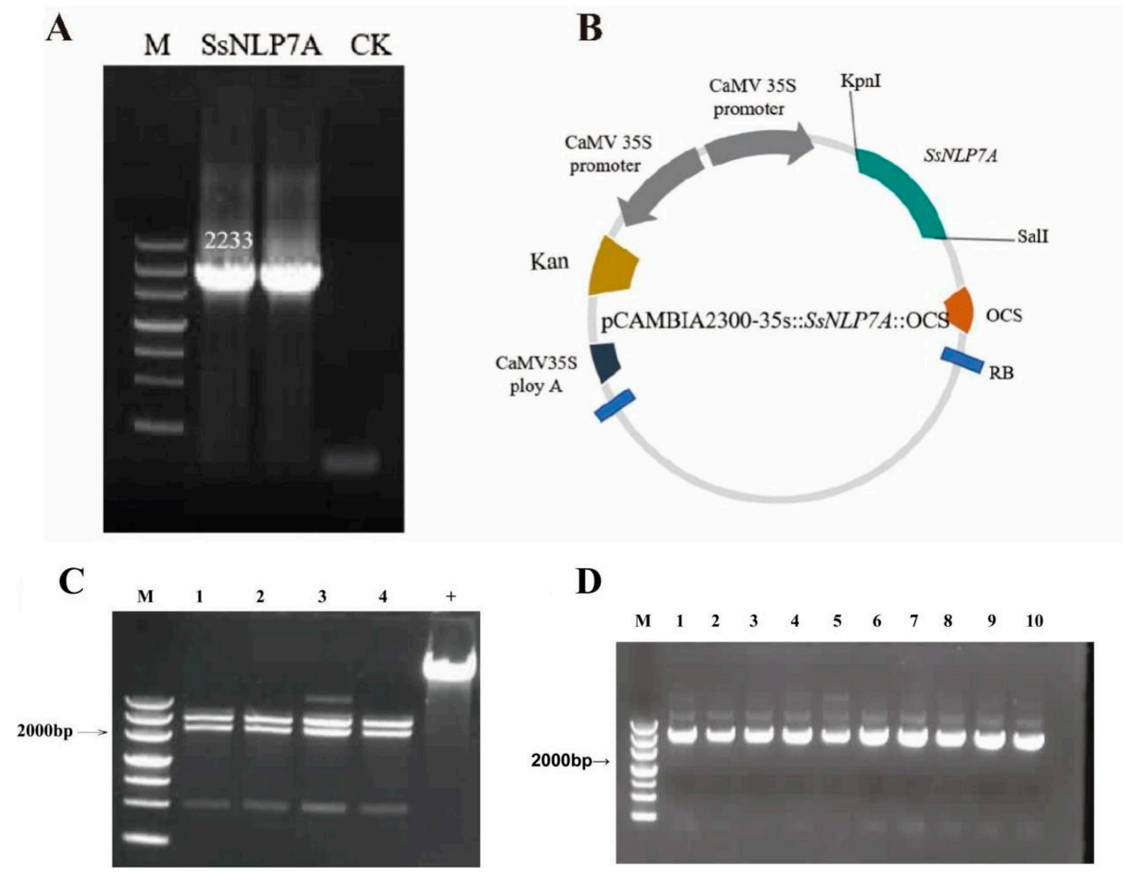

Supplementary Figure S1. (A) Cloning the *SsNLP7A* gene from *Suaeda salsa*. Numbers 1-2 indicate the *SsNLP7A* gene cloned from *Suaeda salsa'*s cDNA. M denotes Maker, CK denotes negative control. (B) *SsNLP7A* overexpression vector map. (C)The identification of recombinant plasmids in plant expression vectors using enzyme digestion. Number 1-4 indicates the use of Kpn I , Sal I and Sal I enzymes to digest the pCAMBIA2300-*SsNLP7A* recombinant plasmid. "+" indicates the plasmid that has not been digested by Kpn I and Sal I double enzymes. "M" indicates the marker (Maker III). (D) Identification of *Agrobacterium tumefaciens* carrying the transformed pCAMBIA2300-*SsNLP7A* recombinant plasmid by PCR. Numbers 1-9 represent the monoclonal samples that were tested. "+" indicates the positive control, "—" indicates the negative control. "M" indicates the marker (Maker III).

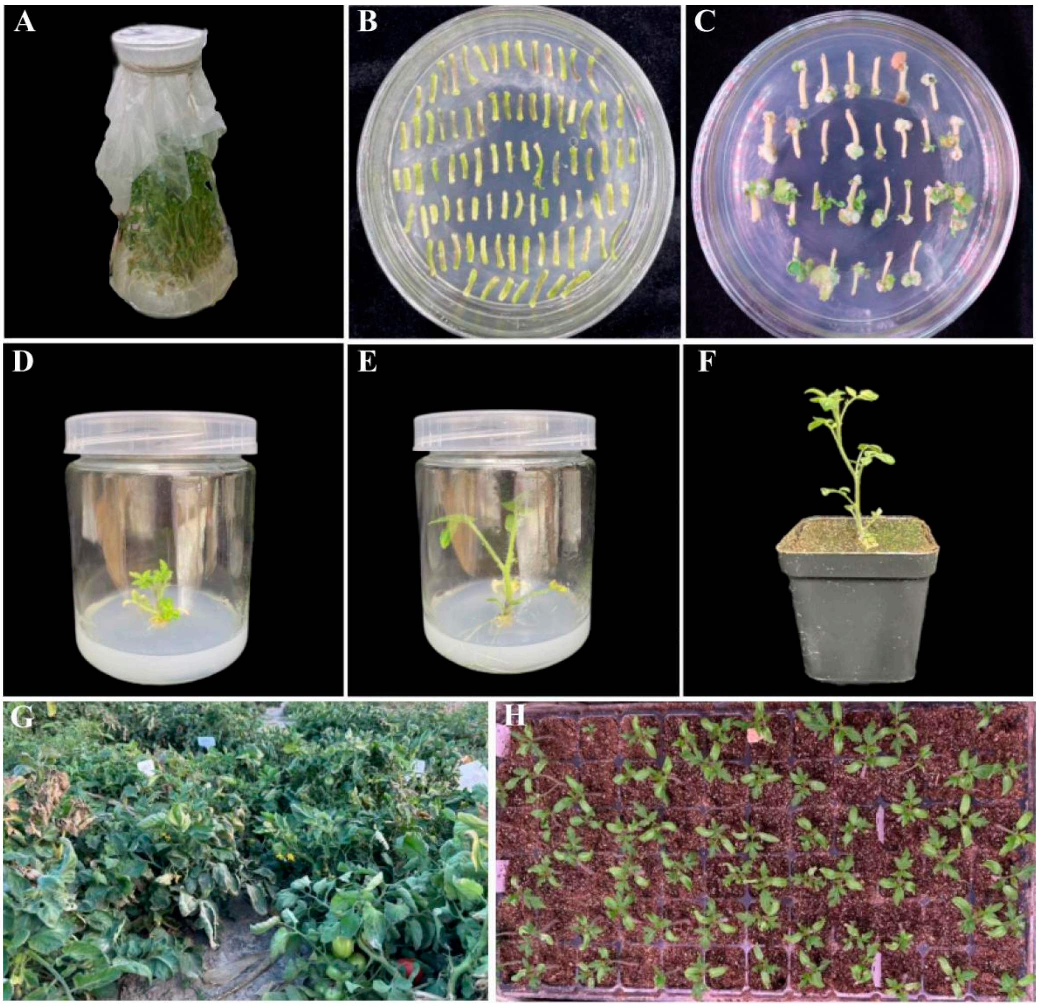

Supplementary Figure S2. The Genetic transformation process of *Solanum lycopersicum*. (B) Callus induced from hypocotyl on MS medium supplemented with 2.0 mg/L 6-BA and 0.5 mg/L NAA. (C) Further differentiation of callus into *Solanum lycopersicum* callus-derived plantlets. (D)Transfer the callus-derived plants to bottles containing culture medium for cultivation.(E) Rooting induction on 1/2MS medium supplemented with 0.5 mg/L IAA. (F) Development of complete plants after transplantation (Pot: 7.2cm inner diameter, 11.5cm high). Bar = 2.5 cm.(G)Transplant plants to the field for cultivation and collect seeds.(H)Harvest T2 generation seeds for laboratory cultivation to prepare for subsequent experiments.

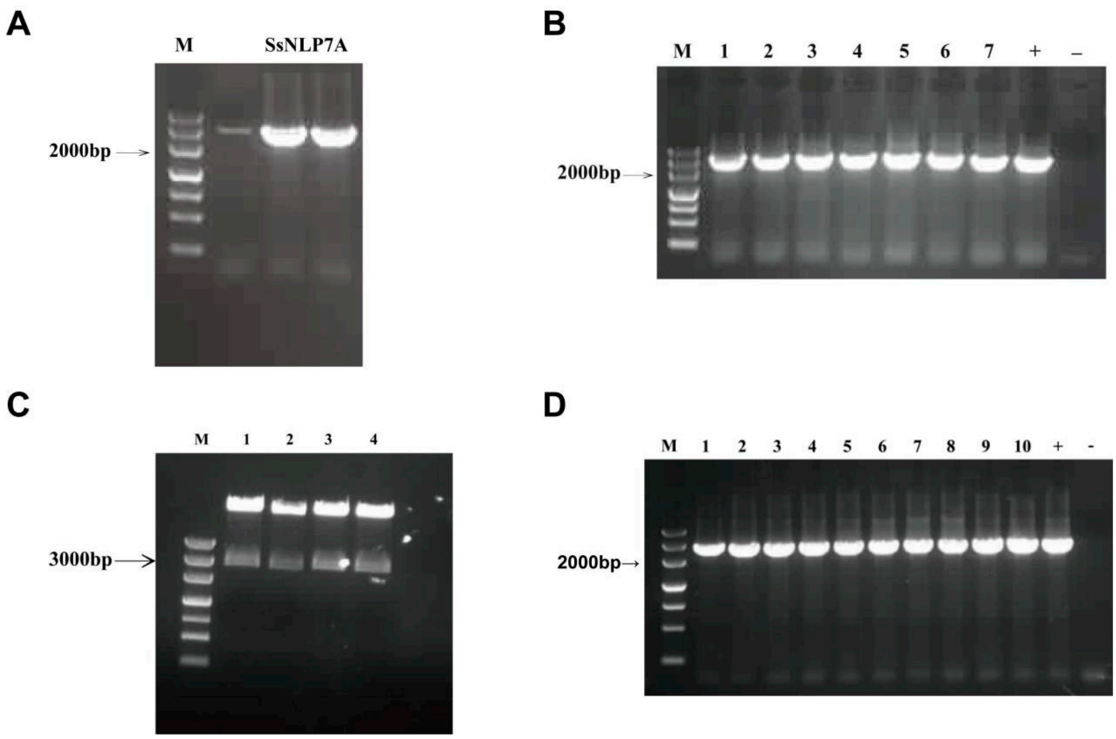

Supplementary Figure S3. Construction of transient expression vectors of *SsNLP7A*.(A)Cloning the *GFP* gene from pCAMBIA1304 vector. Numbers 1-2 indicate the *GFP* gene cloned from pCAMBIA1304 plasmid. (B) (C)The identification of recombinant plasmids in plant expression vectors using enzyme digestion. Number 1-4 indicate the use of BamH I and Kpn I enzymes to digest the pCAMBIA2300-*SsNLP7A-GFP* recombinant plasmid. "+" indicates the plasmid without the addition of BamH I and Kpn I as a control, and "M" indicates the marker (Maker III).(D)Identification of *Agrobacterium tumefaciens* carrying the transformed recombinant plasmid by PCR. Numbers 1-10 represent the monoclonal samples of *Agrobacterium tumefaciens* transformed with pCAMBIA2300-*SsNLP7A-GFP*. "+" indicates the positive control, "–" indicates the negative control. "M" indicates the marker (Maker III).

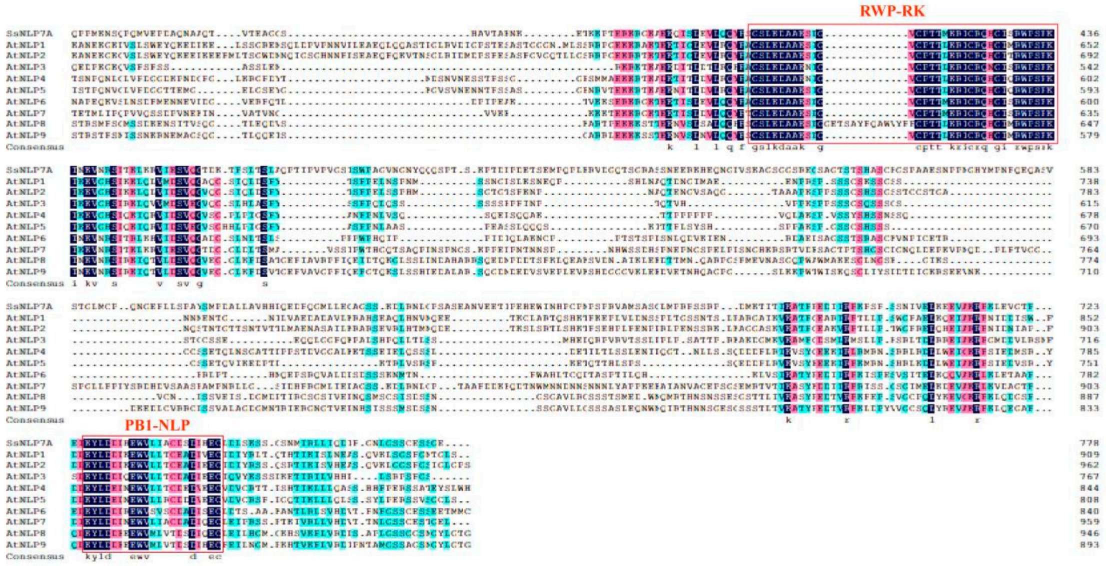

Supplementary Figure S4. *SsNLP7A* sequence structure analysis. Multiple sequence alignment of *SsNLP7A* and NLP7s aquaporin from *Arabidopsis thaliana*.

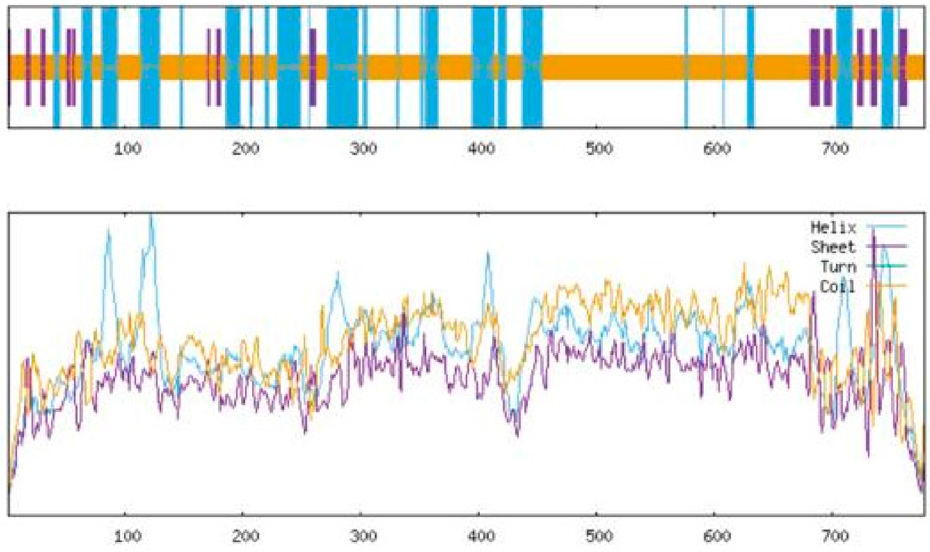

Supplementary Figure S5. Secondary Structure Prediction of SsNLP7A Protein

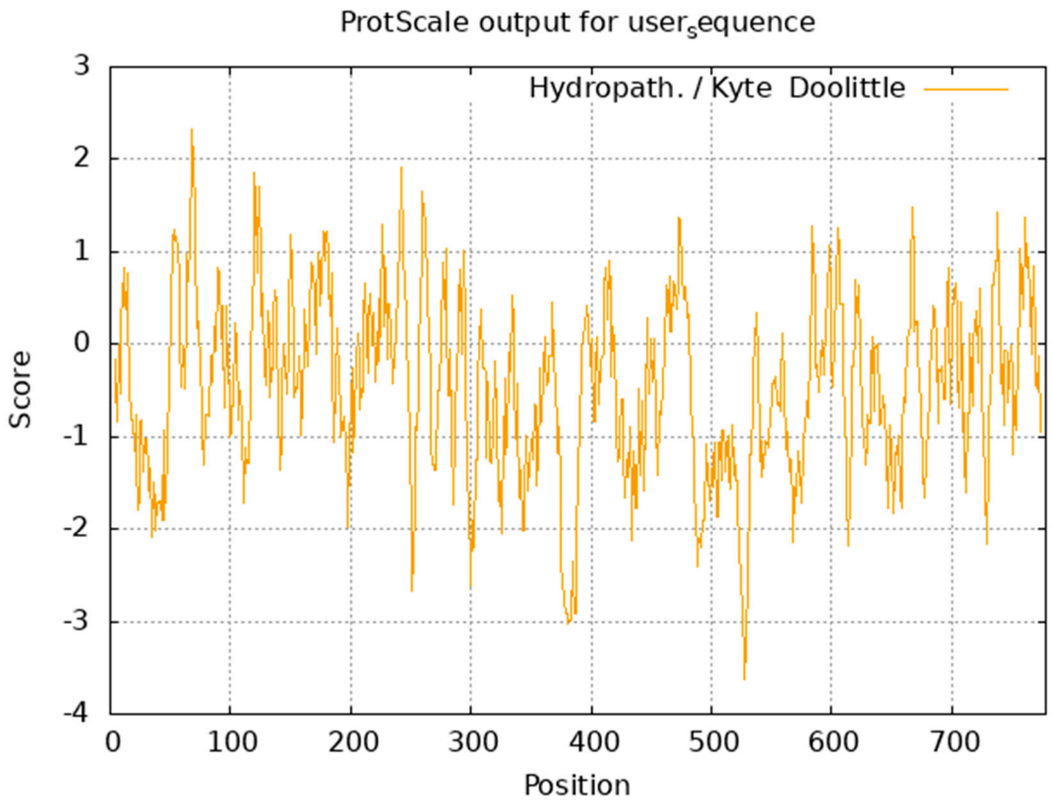

Supplementary Figure S6. SsNLP7A Hydrophilicity/Hydrophobicity Analysis

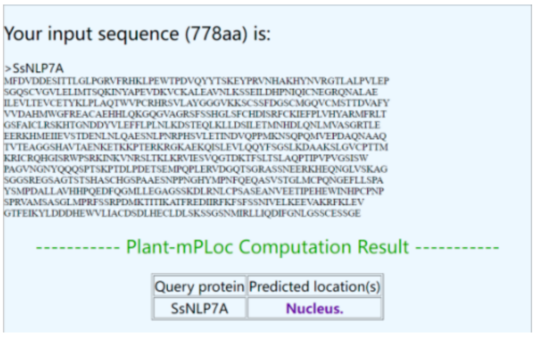

Supplementary Figure S7. Subcellular Localization Prediction for SsNLP7A

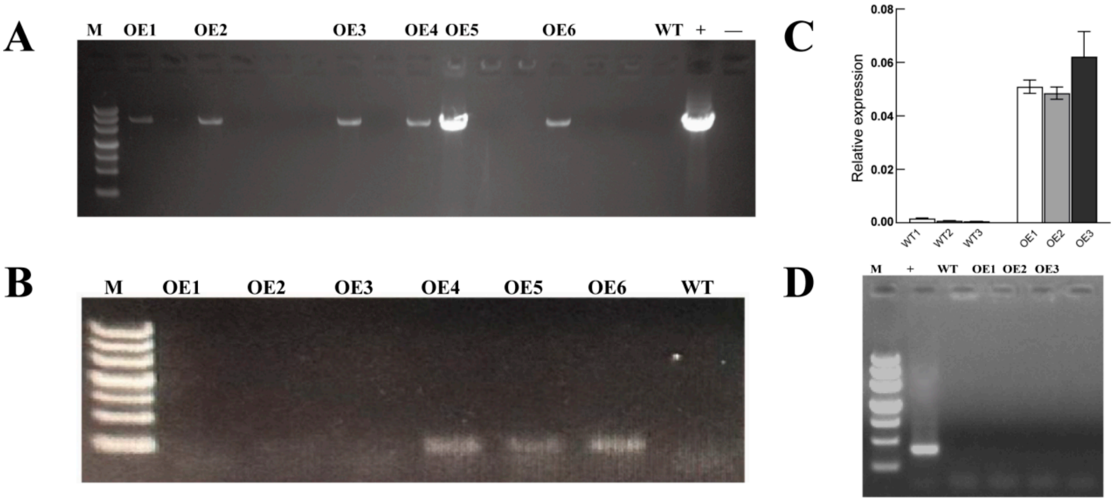

Supplementary Figure S8. Identification of tomato plants overexpressing the *SsNLP7A* gene. (A)Characterization of SsNLP7A transgenic hybrid *Solanum lycopersicum* through DNA-PCR. A total of 15 *Solanum lycopersicum* plants were identified, with 6 plants overexpressing the SsNLP7A gene confirmed as positive. These were designated as OE1, OE2, OE3, OE4, OE5, and OE6. WT represents the wild-type tomato plant. (B) Characterization of SsNLP7A transgenic hybrid *Solanum lycopersicum* through RNA-PCR. Six positive plants identified at the DNA-PCR were subjected to RNA-PCR identification. A total of three positive plants were identified: OE4, OE5, and OE6. WT represents the wild-type *Solanum lycopersicum* plant.(C)cDNA was extracted from leaves of three positive transgenic plants identified from Figure B, alongside cDNA from wild-type tomato leaves. qRT-PCR experiments were conducted using primers listed in Table S1, with wild-type tomato plants serving as controls.The housekeeping gene used is: SlGAPDH. (D) Using genomic DNA from T2-generation transgenic plants, PCR detection was performed with primers specific for the virB2 gene on the *Agrobacterium* Ti plasmid.No specific band for the virB2 gene was amplified in DNA samples from all three transgenic lines (OE1, OE2, OE3) and the wild-type (WT) control, whereas the positive control (Agrobacterium GV3101 genomic DNA) showed a clearly visible band.

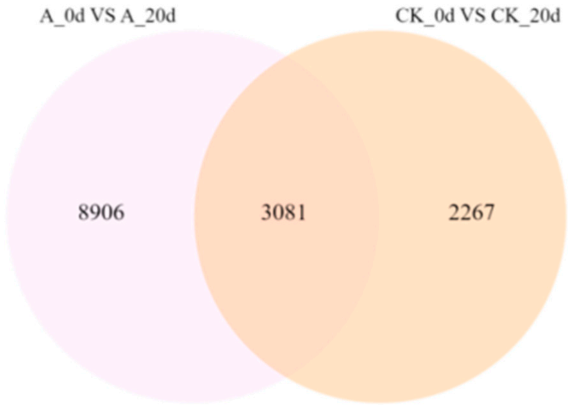

Supplementary Figure S9. Venn diagram of differentially expressed genes between A_0d VS A_20d and CK_0d VS CK_20d groups

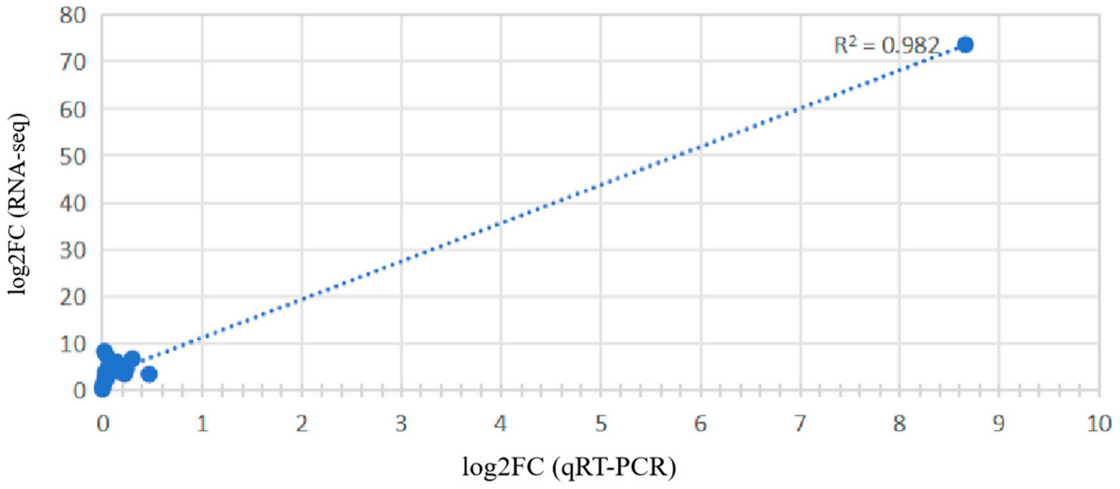

Supplementary Figure S10. Consistency between RNA-seq and qRT-PCR in detecting fold change (FC) in gene expression

Supplementary Tables

Supplementary Table S1 Primer sequences

Primer	Sequence (5'→3')	Use
SsNLP7A-F	cccgggTATGTTTGATGTTGATGATGAGAGC	gene cloning
SsNLP7A-R	gtcgacTCCTCCTGAGCTTTCACACGTAA	gene cloning
VirB2-F	AATAATGCGATGCTTCTCTTC	identification
VirB2-R	AGACGATCATTATTAGCTTGC	identification
GAPDH-F(Q)*	TAGCAAGGATGCTCCCATGTTCGT	qRT-PCR

GAPDH-R(Q)*	AAAGGAGCAAGGCAGTTGGTTGTG	qRT-PCR
SsNLP7D-F(Q)	TGGCTCTATTGCTCTTCCTGTT	qRT-PCR
SsNLP7D-R(Q)	ATTTCTGAACTCCGGAGATGAA	qRT-PCR
SsNLP7A-F(Q)	ATGCTCCTGAGGTTGATAAAGTTT	qRT-PCR
SsNLP7A-R(Q)	TTCCAAAATTTCAGCAAGCG	qRT-PCR
SsNLP7B-F(Q)	TGGGTGTTGTTGACGTGTGA	qRT-PCR
SsNLP7B-F(Q)	TCACAAATGCCCGTTGCTTG	qRT-PCR
SsNLP7C-F(Q)	CAGGGTCCGGGAAAACATCA	qRT-PCR
SsNLP7C-F(Q)	CCAGGACAAGGGAGACCCTA	qRT-PCR
CAT-F(Q)	TGCTTGAAGAACACACTCTCAATTT	qRT-PCR
CAT-R(Q)	CACCTTCCTTGATCTTCTTGTGTAA	qRT-PCR
SOD-F(Q)	CCACCCCTCAAATAGGTCAGT	qRT-PCR
SOD-R(Q)	TCACTGCTGTTAAGGACGGC	qRT-PCR
POD-F(Q)	GGATCTCACACGAGATGCTT	qRT-PCR
POD-R(Q)	ACGACATAACAATGTAACACGC	qRT-PCR
NCED1-F(Q)	TTTTACGCTCGTGGGCTCTT	qRT-PCR
NCED1-F(Q)	GCCGGTGGGTGTTACCTTTA	qRT-PCR
Actin-F(Q)*	CAATCCAAGAGAGGTATCC	qRT-PCR
Actin-F(Q)*	CATTGCTGGAGTGTTGAAGG	qRT-PCR
DREB1-F(Q)	TCCTGGGCAACTACATCTGC	qRT-PCR
DREB1-R(Q)	ATATCCATCGCGGTCCCTTC	qRT-PCR
CIPK8-F(Q)	ATGATGGTGCTGTGGCTGAT	qRT-PCR
CIPK8-R(Q)	AGGAAACCAAGATGGGCAGG	qRT-PCR
CIPK5-F(Q)	TCTGTGAGACCTACCAGCTCA	qRT-PCR
CIPK5-R(Q)	GCTAGTTGGTTGCAATAAATGTCAC	qRT-PCR
MAPKKK16-F(Q)	ACGAGTTCCCCAACAAGCAT	qRT-PCR
MAPKKK16-R(Q)	CTCATCAACCCAGCACCAGT	qRT-PCR
MAPKKK20-F(Q)	GGAGGTGCTCTGTCGGAAAA	qRT-PCR
MAPKKK20-R(Q)	TCGCAGTGAACATAGCCACA	qRT-PCR
NHX5	GTGTCCGAGAGGCCAGACTA	qRT-PCR
NHX5	CTCCAACCCAGTTGCCTACA	qRT-PCR
NHX1	TTTGCTCACAACGCACTCAT	qRT-PCR
NHX1	ATCTTTGCCATGCTGCTGAAC	qRT-PCR
HAK5	TTGATGGAGGGGGAAGGAAA	qRT-PCR
HAK5	AGTTTCATTTGGGAGGAAAAATACA	qRT-PCR

WRKY11-F	GTGATCAGAGTTCCGGCGAT	qRT-PCR
WRKY11-R	ATTCCAGGATCATCGGTGGC	qRT-PCR

* The GADPH and Actin gene were selected as internal references.

Supplementary Table S2

Sample	Raw Reads	Clean Reads	Clean Base(G)	Error Rate(%)	Q20(%)	Q30(%)	GC Content(%)
CK1_0d	49965782	48157854	7.22	0.02	98.63	95.45	43.27
CK2_0d	47471016	45841766	6.88	0.02	98.62	95.46	43.06
CK3_0d	48275296	46558350	6.98	0.02	98.7	95.69	43.14
A1_0d	43330674	41283636	6.19	0.02	98.71	95.72	43.51
A2_0d	49693488	47650678	7.15	0.02	98.66	95.52	43.6
A3_0d	50705744	48885392	7.33	0.02	98.56	95.29	43.61
A1_20d	45342318	43057686	6.46	0.02	98.68	95.63	42.54
A2_20d	44798172	43155974	6.47	0.02	98.58	95.33	42.67
A3_20d	42002730	40020980	6	0.03	97.41	92.64	42.39
CK1_20d	43894722	41606634	6.24	0.02	98.54	95.28	41.9
CK2_20d	45311684	42683386	6.4	0.02	98.5	95.22	42.67
CK3_20d	45428114	42235986	6.34	0.02	98.6	95.47	42.28
